# Parallel Reporter Assays Identify Altered Regulatory Role of rs684232 in Leading to Prostate Cancer Predisposition

**DOI:** 10.3390/ijms22168792

**Published:** 2021-08-16

**Authors:** Naixia Ren, Qingqing Liu, Lingjie Yan, Qilai Huang

**Affiliations:** Shandong Provincial Key Laboratory of Animal Cell and Developmental Biology, School of Life Sciences, Shandong University, Qingdao 266237, China; naixiaren@gmail.com (N.R.); qingqingliu12@gmail.com (Q.L.); yanlingjie32@gmail.com (L.Y.)

**Keywords:** parallel reporter assay, rs684232, FOXA1, *VPS53*, FAM57A, *GEMIN4*

## Abstract

Functional characterization of cancer risk-associated single nucleotide polymorphism (SNP) identified by genome-wide association studies (GWAS) has become a big challenge. To identify the regulatory risk SNPs that can lead to transcriptional misregulation, we performed parallel reporter gene assays with both alleles of 213 prostate cancer risk-associated GWAS SNPs in 22Rv1 cells. We disclosed 32 regulatory SNPs that exhibited different regulatory activities with two alleles. For one of the regulatory SNPs, rs684232, we found that the variation altered chromatin binding of transcription factor FOXA1 on the DNA region and led to aberrant gene expression of *VPS53*, *FAM57A*, and *GEMIN4*, which play vital roles in prostate cancer malignancy. Our findings reveal the roles and underlying mechanism of rs684232 in prostate cancer progression and hold great promise in benefiting prostate cancer patients with prognostic prediction and target therapies.

## 1. Introduction

More than 2000 genome-wide association (GWASs) studies have been published, identifying many loci associated with susceptibility to over 1000 unique traits and common diseases since 2005 [1,2]. Prostate cancer (MIM:176807) is the second most common cancer in males and the fifth leading cause of cancer death in men worldwide [3,4]. As with other complex diseases, the genetic heritability of prostate cancer is caused by both rarely occurring but higher penetrant genetic variants and moderate to commonly occurring variants conferring lower risks. So far, GWAS has identified over 170 low-penetrance prostate cancer susceptibility loci, including more than 1000 SNPs, predominantly in populations of mixed European ancestry [5,6,7,8,9]. Current researches on prostate cancer susceptibility variants can explain 34.4% of the familial risk of prostate cancer, with approximately 6% accounted for by rarely occurring variants and 28.4% attributed to more commonly occurring [minor allele frequency (MAF) > 1%] SNPs as well as some rarer single nucleotide variants [10]. Importantly, a significant number of susceptibility variants have been elucidated for their roles and underlying mechanism in leading to disease susceptibility [11,12,13,14,15,16,17,18,19,20,21,22,23]. Nevertheless, there is still a substantial knowledge gap between SNP-disease associations derived from GWASs and an understanding of how these risk SNPs contribute to the biology of human diseases [24].

A significant challenge remains to identify the functional SNPs from a large number of risk variants. These causal SNPs often locate in gene regulatory elements and can lead to transcriptional misregulation of cancer-related genes [14,15,25]. A parallel reporter gene assay method is urgently needed to evaluate the potential regulatory function of these SNPs. So far, several DNA barcode-based parallel reporter methods have been applied to the screening of regulatory risk sites [26,27,28,29,30,31,32,33,34]. Among them, the dinucleotide reporter system (DiR) was developed to realize parallel reporter assay with minimized tag composition bias, which made it suitable for investigating the subtle regulatory effect from the causal SNPs [34].

In this study, we applied the DiR-seq method to evaluate the prostate cancer risk-related SNPs in 22Rv1 cells. From 213 SNPs, we disclosed 32 regulatory SNPs with their two alleles conferring different regulatory activities. The rs684232 site is one of the regulatory sites and has been widely reported for association with prostate cancer susceptibility in European ancestry men [7,8,23,35,36]. However, the function and mechanism leading to cancer progression still remain unknown. We discovered that the rs684232 T allele increased forkhead box A1 (FOXA1 [MIM:602294]) binding and led to elevated gene expression of *VPS53* subunit of GARP complex (*VPS53* [MIM:615850]), family with sequence similarity 57 member A (*FAM57A* [MIM:611627]), and gem nuclear organelle associated protein 4 (*GEMIN4* [MIM:606969]). The upregulation of the three genes often occurred in prostate cancer tissues and was associated with low disease-specific survival probability for prostate cancer patients. Our findings reveal the roles and underlying mechanism of risk SNP in prostate cancer progression and contribute to defining it as a biomarker for prostate cancer susceptibility or therapeutic responses.

## 2. Results

### 2.1. DiR Assay Discovers Regulatory SNPs in Prostate Cancer Cells

**Figure 1:**
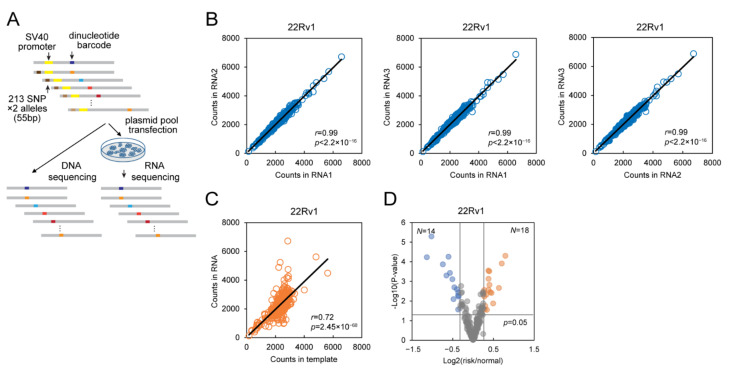
DiR-seq assays discovered regulatory SNPs in prostate cancer cells. (**A**) Flowchart of the DiR system for reporter gene assay in prostate cancer cells. The DiR plasmid library was developed based on the pGL3-promoter vector by optimizing the luciferase coding sequence. The 55 bp SNPs fragment was inserted upstream of the SV40 promoter, 450 bp dinucleotide-barcoded sequences were used as the reporter gene. (**B**) Consistency evaluation between individual biological replicates of DiR-seq assay of 213 prostate cancer risk SNPs in 22Rv1 cells. The correlation coefficient values and *p* values were calculated with Pearson correlation analysis (**C**) Scatter plot of DiR-seq tag counts in RNA and DNA template in 22Rv1 cells. The correlation coefficient values and *p* values were calculated with Pearson correlation analysis. (**D**) Volcano plot of the allelic ratio of reporter expression levels in DiR-seq analysis in the 22Rv1 cell line. *p* values came from a two-tailed Student’s *t*-test of the reporter expression of individual alleles. The blue dots represent SNP sites satisfying the criteria of fold change < 0.8, *p* < 0.05, and the orange dots represent SNP sites satisfying the criteria of fold change > 1.2, *p* < 0.05.

The majority of the SNPs that have associations with increased cancer risk function as potential gene regulatory elements [14,15,25]. Notably, up to 57.1% of GWAS SNPs are located in the DHSs (DNase hypersensitive sites), which indicates that most GWAS SNPs have potential regulatory functions themselves [25]. To identify functional prostate cancer risk-associated variants displaying transcription regulatory function, we used the DiR-seq approach to evaluate the 213 prostate cancer risk SNPs with both risk and protective alleles from the previous GWAS catalog (Table S1). We cloned the 55 bp fragments bearing individual alleles in the middle and inserted them into the DiR vectors right upstream of the basic SV40 promoter (Figure 1A). After transfection of the plasmid pool in the cells, the RNA was extracted, reverse transcribed, prepared next-generation sequencing (NGS) library, and subjected to sequencing (Figure 1A). For all variants, a blanked DiR construct without any insertion was taken as a control. Considering that the typical transcription factors only occupy 6–12 bp DNA sequences [37], and short DNA stretches bearing transcription factor binding sites were widely used in the reporter gene assay [38], the 55 bp fragment will generally be enough to assess the effect of SNPs, even though we might not observe the full activity of the potential herein enhancer element. We used 150-bp paired-end sequencing to ensure the detection of the variants at the DiR barcode sequences (450 bp). Using this method, we could derive the allelic regulatory activities in DNA sequencing compared with those in RNA sequencing.

Our DiR-seq analysis in 22Rv1 cells showed that the tag expression levels had high consistency between individual replicates (Figure 1B), and some SNP sites exhibited elevated tag expression levels compared to the template (Figure 1C). Since the two alleles of the functional SNPs are supposed to drive gene expression differentially, we picked SNPs based on the ratio of reporter expression level from the risk and protective alleles (Figure 1D). In the 22Rv1 prostate cancer cell line, 14 SNPs exhibited decreased expression levels for the risk alleles (risk/protective < 0.8, *p* < 0.05), and 18 SNPs showed increased expression to the contrary (risk/protective > 1.2, *p* < 0.05) (Figure 1D). All of the SNPs picked out in the DiR-seq analysis are listed in Table S6.

### 2.2. Chromatin Status of 32 Functional SNPs

**Figure 2:**
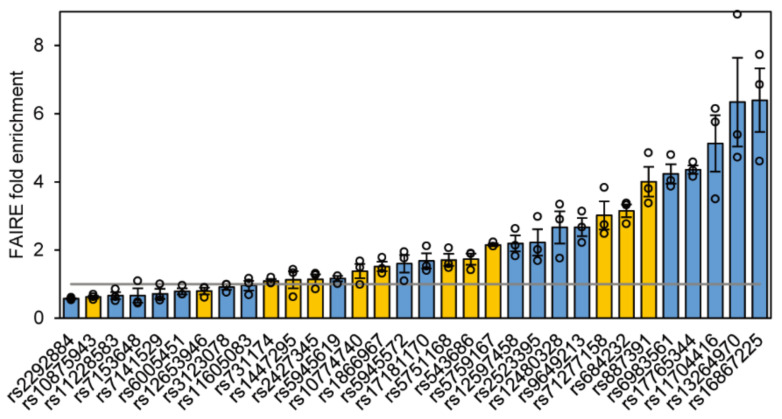
Chromatin status of the 32 functional SNPs in 22Rv1 cells. FAIRE-qPCR analysis of the 32 regulatory SNPs identified by DiR-seq analysis in 22Rv1. Heterozygous SNPs are highlighted in orange. Mean ± SEM of three independent experiments.

In eukaryotes, transcription activating elements usually locate in chromatin regions having high open status. We evaluated the chromatin open status of the 32 regulatory SNPs identified in the 22Rv1 DiR-seq analysis by the FAIRE qPCR method [39] and found more than half significantly enriched in the FAIRE DNA (Figure 2). Among the 32 regulatory SNPs, thirteen heterozygous SNP sites are highlighting in orange in Figure 2. Interestingly, Sanger sequencing chromatography of the FAIRE DNA showed strongly allele-specific openness for rs684232, rs887391, and rs5759167 sites in 22Rv1 cells (Figure S1A,B and S2A,B). We then chose the two most enriched heterozygous sites, rs684232 and rs887391, for further exploration.

### 2.3. Allele-Specific Activity of rs684232 and rs887391

**Figure 3:**
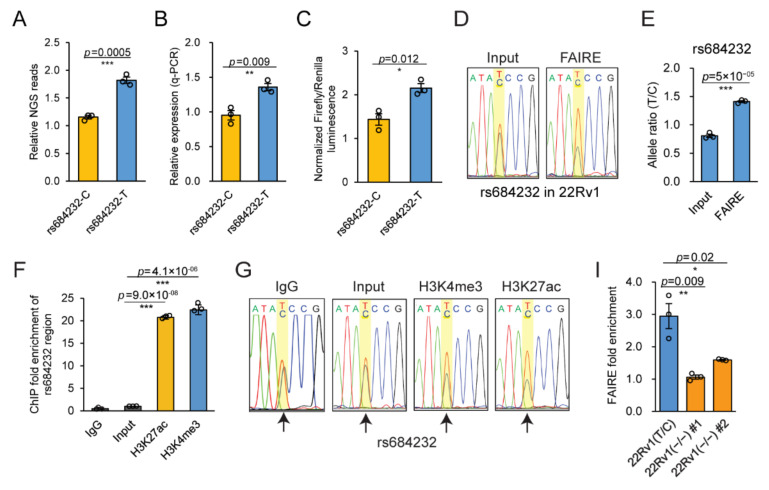

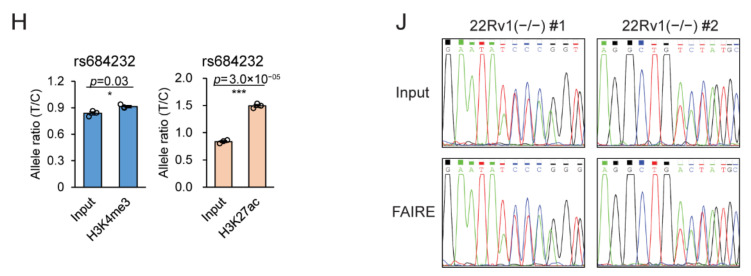
Chromatin status of the functional SNP rs684232 in 22Rv1 cells. (**A**) Reporter gene expression level for rs684232 SNP region in the DiR-seq analysis in 22Rv1 cells. T allele showed increased enhancer activity relative to the C allele. Mean ± SEM of three independent experiments. *** *p* < 0.001, two-tailed Student’s *t*-test. (**B**) Reporter gene expression level for rs684232 SNP region in the DiR-qPCR assay in 22Rv1 cells. Mean ± SEM of three independent experiments. ** *p* < 0.01, two-tailed Student’s *t*-test. (**C**) Normalized luciferase activity for the rs684232 region in 22Rv1 cells. T allele showed increased enhancer activity relative to the C allele. Mean ± SEM of three independent experiments. * *p* < 0.05, two-tailed Student’s *t*-test. (**D**) Sanger sequencing chromatography of FAIRE DNA for the rs684232 site in 22Rv1. The position of rs684232 is highlighted in a yellow square. (**E**) Allele-specific enrichment of rs684232 site in FAIRE DNA determined in 22Rv1 cells by AS-qPCR. Mean ± SEM of three independent experiments. *** *p* < 0.001, two-tailed Student’s *t*-test. (**F**) Fold enrichment of the rs684232 region in H3K27ac and H3K4me3 ChIP DNA in 22Rv1. Nonspecific immunoglobulin G (IgG) and Input as the negative control. Mean ± SD of three technical replicates. *** *p* < 0.001, two-tailed Student’s *t*-test. (**G**) Sanger sequencing chromatography of rs684232 in H3K27ac and H3K4me3 ChIP DNA in 22Rv1 cells. IgG and Input as the negative control. The position of rs684232 is highlighted in a yellow square. (**H**) Allele-specific enrichment of rs684232 region in ChIP DNA of H3K27ac and H3K4me3 modification determined by AS-qPCR in 22Rv1. Mean ± SD of three technical replicates. * *p* < 0.05, *** *p* < 0.001, two-tailed Student’s *t*-test. (**I**) Chromatin open status analysis of the rs684232 region in the two genome-edited cell lines 22Rv1(−/−) #1 and 22Rv1(−/−) #2. Mean ± SEM of three independent experiments. * *p* < 0.05, ** *p* < 0.01, two-tailed Student’s *t*-test. (**J**) Sanger sequencing chromatography of the FAIRE DNA around rs684232 sites in the two genome-edited 22Rv1 cell lines.

In 22Rv1 cells, DiR-seq (Figure 3A), DiR-qPCR (Figure 3B), and luciferase reporter assays (Figure 3C) indicated that the rs684232 region could drive reporter expression, and the T allele exhibited significantly higher activity than the C allele. Interestingly, the T allele was also highly preferred in the active chromatin region, as shown in Sanger sequencing chromatography of the FAIRE DNA (Figure 3D), and the allele-specific enrichment was also confirmed by AS-qPCR (Figure 3E). For the site rs887391, the T allele exhibited significantly higher enrichment than the C allele in FAIRE DNA as determined by AS-qPCR (Figure S2C). Besides, the rs11672691 site, one SNP in LD (Linkage disequilibrium) with rs887391, also enriched in the FAIRE DNA in an allele-specific manner (Figure S2D–F), whose biological function and underlying mechanism had been illustrated previously [15,40].

The H3K4me3 and H3K27ac histone modifications are usually the markers of active gene regulatory elements [14,41,42]. Our ChIP qPCR analysis showed that rs684232 (Figure 3F), rs887391, and rs11672691 (Figure S2G) were significantly enriched in these two types of histone modifications. Interestingly, the enrichment of the rs684232 site in histone modifications also displayed a strong preference for the T allele (Figure 3G). Further AS-qPCR analysis of the ChIP DNA confirmed the allele-specific enrichment (Figure 3H). Correspondingly, both rs887391 and rs11672691 also exhibited allele-specific enrichment in the H3K4me3 and H3K27ac ChIP DNA (Figure S2H–K). The results suggest that all three risk SNPs are potential gene regulatory variants. Since the rs11672691 and rs887391 have been reported previously for their biological function and underlying mechanism [15,40], we focused on the rs684232 site in the subsequent mechanism study.

### 2.4. The Gene Regulatory Function of SNP rs684232

The rs684232 site has been reported for association with prostate cancer susceptibility [7,8,36] and determined to be an important expression quantitative trait locus (eQTL) [42,43,44,45]. To further investigate the potential function of rs684232, we obtained two rs684232-edited single-cell clones, named 22Rv1(−/−) #1 and 22Rv1(−/−) #2 (Figure S3), through the CRISPR/Cas9 technology. Notably, indel mutation of the rs684232 element in the edited 22Rv1 cell clones led to hindered chromatin openness of this SNP region as evaluated by FAIRE qPCR analysis (Figure 3I). The Sanger sequencing chromatography of FAIRE DNA indicated that the allele preference also diminished upon the genome editing (Figure 3J).

The rs684232 site locates in 17p13.3 loci, 2 kb upstream of gene *VPS53*, and its LD SNPs rs2955626 and rs461251 were proposed to be the possible functional variants [7]. In our FAIRE analysis in 22Rv1, even though both SNPs were significantly enriched in the open chromatin regions (Figure S4A), neither exhibited significant allele preference as evaluated with Sanger sequencing and AS-qPCR analysis (Figure S4B–G). Besides, both SNPs were also enriched significantly in the H3K4me3 and H3K27ac histone modification regions in ChIP analysis (Figure S4H,I). Nevertheless, rs2955626, the higher enrichment site, did not exhibit allele specificity in both histone modifications as determined in Sanger sequencing and AS-qPCR analysis (Figure S4J,K), and rs461251 displayed allele specificity only for the H3K27ac modification (Figure S4L,M). In the luciferase reporter assay, the rs2955626 site did not exhibit apparent regulatory activity (Figure S5A). Even though the rs461251 genomic region showed apparent reporter gene activity, the two alleles did not drive gene expression differentially. (Figure S5B). It indicated that rs684232 should be the causal SNP on this locus, and the allele preference of rs461251 in H3K27ac modification might attribute to their closeness to the rs684232 site.

### 2.5. rs684232 Affects FOXA1 Chromatin Binding

**Figure 4:**
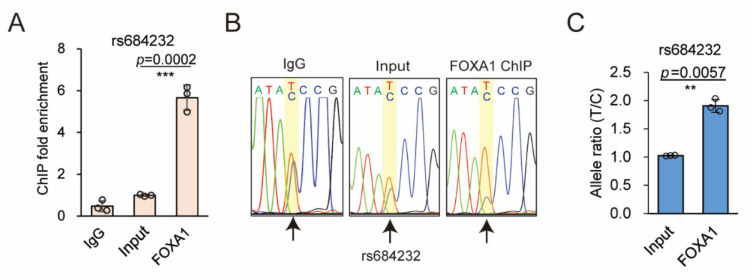
SNP rs684232 alters FOXA1 binding. (**A**) Enrichment of rs684232 region in the FOXA1 ChIP DNA in 22Rv1 cells. IgG and Input as the negative control. Mean ± SD of three technical replicates. *** *p* < 0.001, two−tailed Student’s *t*-test. (**B**) Sanger sequencing chromatography of FOXA1 ChIP DNA for the rs684232 site in 22Rv1 cells. IgG and Input as the negative control. The position of rs684232 is highlighted with a yellow square and arrow. (**C**) Allele−specific enrichment of rs684232 site in the FOXA1 ChIP DNA determined by AS−qPCR in 22Rv1 cells. Mean ± SD of three technical replicates. ** *p* < 0.01, two−tailed Student’s *t*-test.

Next, we explored the potential transcription factors that participate in the biological function of the rs684232 site using the HaploReg v4.1 [46]. We found that FOXA1 was the potential transcription factor that bound the rs684232 region. Our ChIP qPCR analysis further showed that the rs684232 region was significantly enriched in the FOXA1 cistrome in 22Rv1 cells (Figure 4A). Notably, the T allele was significantly preferred for the FOXA1 chromatin binding, as shown in the Sanger sequencing (Figure 4B) and AS-qPCR assay (Figure 4C). These results indicate that the rs684232 might affect the chromatin binding of FOXA1.

### 2.6. rs684232 Regulates Gene Expression of VPS53, FAM57A, and GEMIN4 through FOXA1

**Figure 5:**
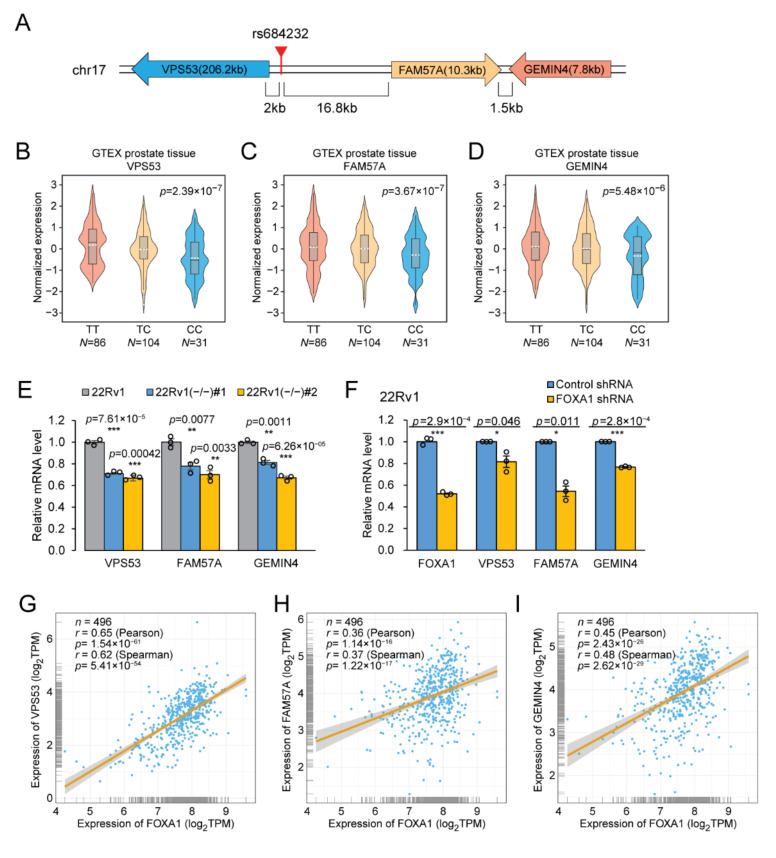

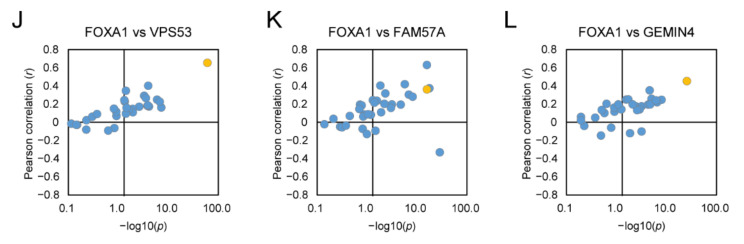
rs684232 regulates gene expression of *VPS53*, *FAM57A*, and *GEMIN4* through FOXA1. (**A**) The location of the rs684232 relative to three target genes. (**B**–**D**) eQTL analysis in GTEx prostate tissues to reveal the association between alleles of rs684232 and *VPS53* (**B**), *FAM57A* (**C**), and *GEMIN4* (**D**) genes. *p* values are from a linear regression model. (**E**) Gene expression quantification of *VPS53, FAM57A*, and *GEMIN4* by RT-qPCR in three 22Rv1 cells, including parental 22Rv1 cells and mutated cells 22Rv1(−/−) #1/ #2. Mean ± SEM of three biological replicates. ** *p* < 0.01, *** *p* < 0.001, two-tailed Student’s *t*-test. (**F**) Gene expression quantification of *VPS53, FAM57A,* and *GEMIN4* by RT-qPCR in 22Rv1 cells treated with *FOXA1* shRNA. Mean ± SD of three technical replicates. * *p* < 0.05, *** *p* < 0.001, two-tailed Student’s *t*-test. (**G**–**I**) Gene expression correlation analysis between transcription factor FOXA1 and the three target genes *VPS53* (**G**), *FAN57A* (**H**), and *GEMIN4* (**I**) in prostate tumor tissues from TCGA-PRAD database. Correlation coefficient (*r*) values and *p* values were from Pearson or Spearman correlation analysis, respectively. (**J**–**L**) Gene expression correlation analysis between FOXA1 and *VPS53* (**J**), *FAM57A* (**K**), or *GEMIN4* (**L**) in 33 kinds of cancer tissues from TCGA. The Pearson correlation coefficient value of each cancer type was plotted vs. the −log10 of *p*-value. The dot in yellow represents the PRAD. Correlation coefficient (*r*) values and *p* values were from the Pearson correlation analysis.

The rs684232 site locates in 17p13.3 loci accompanied by three nearby genes, *VPS53*, *FAM57A*, and *GEMIN4* (Figure 5A). In eQTL analysis using the genotype-tissue expression (GTEx) database, *VPS53*, *FAM57A*, and *GEMIN4* genes all exhibited significant associations with the rs684232 variation by the normalized effect size (NES) of −0.40, −0.27, and −0.26, respectively (Figure 5B–D). Notably, the T allele, which was preferred in the active chromatin and exhibited higher activity in the reporter gene assay, also corresponded to higher expression levels for all three genes. We also found that the expression level of the three target genes significantly decreased upon indel mutation of the rs684232 site in 22Rv1(−/−) #1 and 22Rv1(−/−) #2 cells (Figure 5E). Furthermore, as the causal SNP, the heterozygous rs684232 site should drive allele-specific expression of the three genes in 22Rv1 cells. To investigate the allele imbalance, we picked three heterozygous SNPs, rs11558129, rs113201579, and rs3744741, in the exon of *VPS53*, *FAM57A*, and *GEMIN4*, respectively. Sanger sequencing chromatography of the 22Rv1 cDNA showed that all three genes had allele-specific expression (Figure S6A). Remarkably, when the rs684232 were mutated by indel, the allele preference in all the three genes diminished accordingly (Figure S6A). Moreover, FOXA1 knockdown with shRNA led to significant down-regulation of *VPS53*, *FAM57A*, and *GEMIN4* genes in 22Rv1 cells (Figure 5F). The results indicate that the rs684232 site might regulate gene expression of *VPS53*, *FAM57A*, and *GEMIN4* by affecting FOXA1 binding.

To further explore the relationship between FOXA1 and the three target genes, *VPS53*, *FAM57A*, and *GEMIN4*, we performed a gene express association assay using RNA-seq data from The Cancer Genome Atlas (TCGA, The Cancer Genome Atlas, RRID:SCR_003193) prostate cancer tissues (TCGA-PRAD, dbGaP Study Accession: phs000178). We found that the mRNA level of FOXA1 positively correlated strongly with *VPS53* (Spearman correlation coefficient = 0.62, Figure 5G), weakly with *FAM57A* (Spearman r = 0.37, Figure 5H), and moderately with *GEMIN4* (Spearman r = 0.48, Figure 5I) genes. Interestingly, for the 33 cancer types from the TCGA Pan-Cancer analysis project, up to 19, 16, and 16 cancer types displayed positive correlations for *FOXA1* vs. *VPS53, FOXA1* vs. *FAM57A*, and *FOXA1* vs. *GEMIN4*, respectively (Figure 5J–L). These results provide evidence that transcription factor FOXA1 regulates gene expression of *VPS53*, *FAM57A*, and *GEMIN4*.

Surprisingly, we also observed that the three target genes strongly correlated with each other in TCGA prostate cancer tissues with a Pearson correlation coefficient of 0.61, 0.7, and 0.78, respectively (Figure S6B). When explored in the TCGA Pan-Cancer tissues, all the 33 cancer types displayed a positive pairwise correlation between *VPS53*, *FAM57A*, and *GEMIN4* (Figure S6C). The results indicate that the three target genes *VPS53*, *FAM57A*, and *GEMIN4* may have a very important regulatory role in prostate cancer disease, and there may also be a synergistic promoting effect between them.

### 2.7. VPS53, FAM57A, and GEMIN4 Knockdown Impedes Cancerous Phenotypes

**Figure 6:**
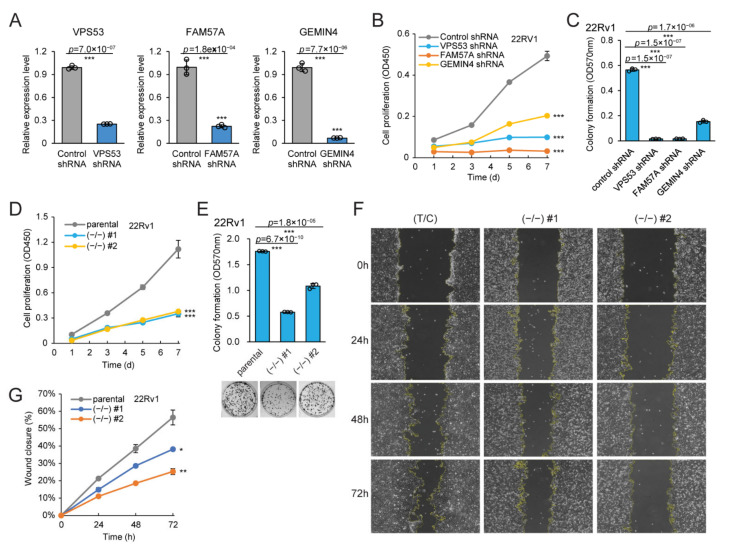
*VPS53*, *FAM57A*, and *GEMIN4* knockdown impede cancerous phenotypes. (**A**) Effect evaluation of lentiviral shRNAs targeting *VPS53*, *FAM57A*, and *GEMIN4* in 22Rv1 cells. Mean ± SD of three technical replicates. *** *p* < 0.001, two-tailed Student’s *t*-test. (**B**) Cell proliferation assay of 22Rv1 cells undergoing lentiviral shRNA gene knockdown targeting *VPS53, FAM57A*, and *GEMIN4*, respectively. Cell viability was determined using the CCK-8 method at 1–7 d post-seeding. Mean ± SD of three biological replicates. *** *p* < 0.001, two-tailed Student’s *t*-test. (**C**) Colony formation assay of 22Rv1 cells undergoing lentiviral shRNA gene knockdown targeting *VPS53*, *FAM57A*, and *GEMIN4*. Cell colonies were quantified through the Crystal Violet staining method. Mean ± SD of three biological replicates. *** *p* < 0.001, two-tailed Student’s *t*-test. (**D**) Cell proliferation assay for the two genome-edited 22Rv1 cells with rs684232 site mutated through indels. Cell viability was determined using the CCK-8 method at 1–7 d post-seeding. Mean ± SD of three biological replicates. *** *p* < 0.001, two-tailed Student’s *t*-test. (**E**) Colony formation assay for the two genome-edited 22Rv1 cells. Cell colonies were quantified through the Crystal Violet staining method. Representative images from triplicate experiments on the bottom. Mean ± SD of three biological replicates. *** *p* < 0.001, two-tailed Student’s *t*-test. (**F**) Wound healing assay of the two rs684232 knockout 22Rv1 cell lines. Representative images from triplicate experiments. (**G**) The wound closure percentages in the wound healing assay experiments were quantified using Image J software. Mean ± SEM of three biological replicates. * *p* < 0.05, ** *p* < 0.01, two-tailed Student’s *t*-test.

To further understand the biological function of rs684232, we first assessed the effect of the *VPS53*, *FAM57A*, and *GEMIN4* genes on cancerous phenotypes in 22Rv1 cells. We found that the lentiviral shRNA knockdown for the three individual genes all impeded cell proliferation dramatically in a time-course CCK-8 assay (Figure 6A, B). Additionally, their capabilities in forming single-cell colonies were also hindered upon gene downregulation (Figure 6C). The results indicate that all three rs684232 target genes, *VPS53*, *FAM57A*, and *GEMIN4*, play essential roles during cell proliferation and colony formation of prostate cancer cells.

Next, we evaluated the cancerous phenotypes of the two genome-edited 22Rv1 cell lines, 22Rv1(−/−) #1 and 22Rv1(−/−) #2, that had rs6842323 site mutated. Remarkably, the genome-edited 22Rv1 cells exhibited decreased capabilities for both cell proliferation (Figure 6D) and colony formation (Figure 6E) dramatically. What is more, the mutation of rs684232 elements also delayed cancer cell migration dramatically, as demonstrated in the wound healing assay (Figure 6F,G). The results indicate that the rs684232 element is vital for cancer malignancy.

### 2.8. VPS53, FAM57A, and GEMIN4 Affect Cancer Progression

**Figure 7:**
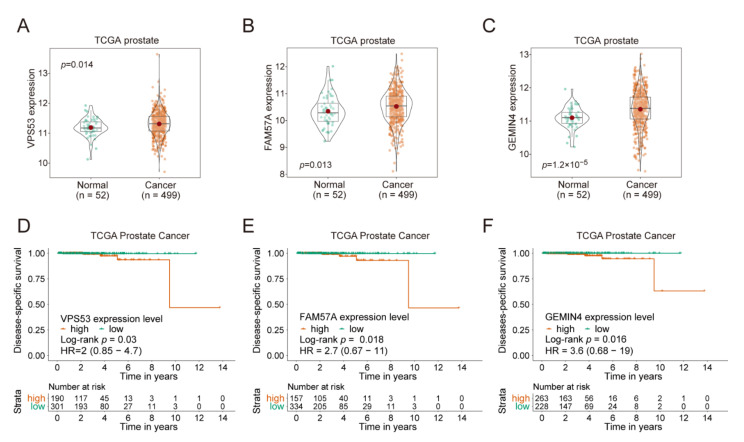
*VPS53*, *FAM57A*, and *GEMIN4* affect cancer progression. (**A**–**C**) Violin box plot to compare gene expression of *VPS53* (**A**), *FAM57A* (**B**), and *GEMIN4* (**C**) at mRNA level between normal and cancer tissues from TCGA. Gene expression value was showed as log2 value of reads data, with the mean, median, 0.25, and 0.75 quantiles represented. *p* values were examined by Mann-Whitney U tests. (**D**–**F**) Kaplan–Meier disease-specific survival analysis of prostate cancer patients that were stratified into two groups according to the expression level of *VPS53* (strata point = 2.52) (**D**), *FAM57A* (strata point = 3.92) (**E**), and *GEMIN4* (strata point = 3.06) (**F**). *p* values were calculated by the log-rank test, with a 95% confidence interval.

We then investigated the expression level of the three rs684232 target genes, *VPS53*, *FAM57A*, and *GEMIN4*, in cancer tissues and their effect on the clinical prognosis of cancer patients. In the TCGA prostate cohort, cancer tissues displayed significantly higher expression levels for the *VPS53* (*p* = 0.014, Figure 7A), *FAM57A* (*p* = 0.013, Figure 7B), and *GEMIN4* (*p* = 1.2 × 10^−5^, Figure 7C) genes in comparison to adjacent normal tissues. Furthermore, we performed the Kaplan–Meier survival analysis for the three genes using the TCGA prostate cancer cohort (Figure 7D–F). We found that patients with higher expression levels of *VPS53* (Log-rank *p* = 0.03, Figure 7D), *FAM57A* (Log-rank *p* = 0.018, Figure 7E), and *GEMIN4* (Log-rank *p* = 0.016, Figure 7F) had worse prostate cancer-specific survival probability. Remarkably, the TCGA Pan-Cancer patients with a higher expression level of *VPS53*, *FAM57A*, and *GEMIN4* also displayed decreased overall survival probability (Figure S7).

We next explored how the three genes affect the disease recurrence. Interestingly we found that patients with lower expression levels of *VPS53* (Log-rank *p* = 0.031) and *GEMIN4* (Log-rank *p* = 0.017) had shorter disease-free intervals (Figure S8). A similar trend was observed with the *FAM57A* gene (Log-rank *p* = 0.109), but without reaching statistical significance.

In brief, the results indicate that the rs684232 site and the target genes *VPS53*, *FAM57A*, and *GEMIN4* are positively associated with prostate cancer cell malignancy, and the high expression for the three genes are associated with poor prognosis for cancer patients.

## 3. Discussion

Risk SNPs have become a hot spot in the cancer research field with the advent of the post-GWAS era [7,47,48]. The DiR-seq screening system has high accuracy and is suitable for functional screening of the risk SNPs, which usually have a modest impact [33,34]. In this study, we applied the DiR system in prostate cancer cells to screen the causal risk SNPs that possess potential gene regulatory functions. We identified 32 regulatory SNPs based on the ratio of reporter expression level from the risk and normal alleles. Among them, fourteen SNPs exhibited decreased expression levels for the risk alleles, and eighteen SNPs showed increased expression to the contrary. The results provide valuable clues to further mechanism elucidation of the functional prostate cancer risk SNPs. However, since only 55 bp SNP site-centered genomic regions were used in our reporter gene assays, an eQTL will be missed if its function involves a larger genomic region or interactions with other molecules bound on distal enhancer sites.

In addition, we disclosed the regulatory pathway for rs684232 sites, in which the SNP site altered the chromatin binding of FOXA1 and led to the misregulated expression of *VPS53*, *FAM57A*, and *GEMIN4*. Notably, mutating the rs684232 element through genome editing or knockdown the expression of *VPS53*, *FAM57A*, and *GEMIN4* genes led to impeded cancer malignancy of 22Rv1 cells. Patients with higher expression of *VPS53*, *FAM57A*, and *GEMIN4* exhibited worse disease-specific survival probability, as demonstrated in the Kaplan–Meier survival analysis on the TCGA prostate cancer cohort. Remarkably, all three target genes were upregulated significantly in tumor tissues compared to adjacent normal tissues in TCGA prostate clinical samples.

Interestingly, we also found that downregulation of *VPS53*, *GEMIN4*, and *FAM57A* genes led to shorter disease-free intervals for prostate cancer patients to the contrary. Similarly, Ramanand et al. [45] recently reported that the impeded expression of *VPS53*, *FAM57A*, and *GEMIN4* genes might cause the increased biochemical recurrence risk for prostate cancer patients. We think that the rs684232 site and its target genes are supposed to be multifaceted in prostate cancer. In prostate cancer patients, the T allele of rs684232 leads to elevated expression of the target genes and is associated with worse disease-specific survival probability. However, when it turns to cancer susceptibility to cancer incidence and the risk for recurrence, the C allele might lead to higher prostate cancer susceptibility on the contrary. Even though complicated to understand, disclosing the seemingly opposite effect for the risk SNP and the three genes is crucial for understanding their biological functions, especially in translational medicine. Otherwise, cancer patients might receive the wrong suggestions and administrations based on one-sided knowledge, and lead to undesirable consequences. However, it is still unclear why that might be, and further systematic investigation of the functions of the three genes is necessary to address this question. The results potentially highlight the complexity of genetic susceptibility to cancer, and more works involving multiple variants and other factors are demanded to fully understand their contribution to cancer susceptibility.

FOXA1 encodes a pioneer factor that induces open chromatin conformation to allow the binding of other transcription factors. FOXA1 has been proved as a driver of prostate cancer onset and progression [49,50,51,52]. The transcription factor FOXA1 has been proven to regulate transcriptional programs in both normal prostate tissue and cancer tissues by directly interacting with AR [53,54,55]. Therefore, other factors such as AR might also participate in the function of rs684232. So, in the future, more depth researches on transcription factor FOXA1 in prostate cancer are needed.

The *VPS53* gene encodes the VPS53 subunit of the GARP complex that functions in retrograde transport from endosomes to the trans-Golgi network (TGN). The *FAM57A* gene encodes a membrane-associated protein that might involve in amino acid transport and glutathione metabolism. The *GEMIN4* gene product is s part of the Gemini bodies that function in spliceosome snRNP assembly and spliceosome regeneration required for pre-mRNA splicing. However, the roles of all three genes in cancer progression remain entirely unknown. To illustrate their functions and underlying mechanisms in affecting cancer susceptibility will be essential topics in the future and give vital clinical implications and translational value for cancer patients.

In general, we identified regulatory prostate cancer risk SNPs by DiR-seq analysis in prostate cancer cell lines and elucidated the function and mechanism of rs684232 in leading to prostate cancer progression. The results described here should be valuable for accurate prognostic prediction of prostate cancer patients in clinical. Further studies on mouse models and clinical samples might be demanded before applied in translational medicine.

## 4. Materials and Methods

### 4.1. Construction of the DiR Reporter Pool for Prostate Cancer Risk SNPs

Prostate cancer risk SNPs list were obtained from the GWAS Catalog (https://www.ebi.ac.uk/gwas/, accessed on 1 August 2016) in 2016, which contained 213 prostate cancer risk SNPs (Table S1) at that time. They are tag SNPs reported in the previous GWAS studies and are significant associated with prostate cancer risk (*p*-value < 10^−5^). We obtained the 55 bp SNP-centered DNA region sequence for both protective and risk alleles from the UCSC genome browser on GRCh38/hg38. The annealed oligos (Table S2) were inserted into the DiR vectors between SmaI and BglII sites using T4 DNA Ligase (EL0011, Thermo Scientific, Waltham, MA, USA) as described previously [34]. DiR constructs were confirmed correct through Sanger sequencing. The 426 reporter constructs for 213 SNPs were mixed with the DiR-Promoter and the DiR-Control vector and then subjected to reporter assays in prostate cancer cells.

### 4.2. Cell Culture

The 22Rv1 (ATCC Cat# CRL-2505, RRID:CVCL_1045) cells used in this study were purchased from the American Type Culture Collection (ATCC) and grown in RPMI-1640 (Gibco, New York, NY, USA) supplied with 10% FBS (Gibco, New York, NY, USA) and 1% antibiotics (Penicillin-Streptomycin, Sigma, St. Louis, MO, USA). The Lenti-X 293T cells were purchased from Clontech Laboratories (Clontech, CA, USA) and maintained in DMEM (Gibco, New York, NY, USA) supplied with 10% FBS (Gibco, New York, NY, USA) and 1% Penicillin-Streptomycin. The cells were cultured at 37 °C with 95% air and 5% CO_2_ and routinely confirmed to be mycoplasma free using the Myco-Blue Mycoplasma Detector (D101-01, Vazyme, Nanjing, China). 22Rv1 cells used in our study were cultured following the ATCC instructions.

### 4.3. Cell Transfection

Plasmids were transfected cells using Lipofectamine 2000 Reagent (11668-019, Invitrogen, Carlsbad, CA, USA) or Polyethylenimine (PEI, 408727-sigma, St. Louis, MO, USA) dependent on cell types. Lipofectamine 2000 was used for 22Rv1, and PEI was used for Lenti-X 293T cells following the manufacturer’s instructions. Cell transfections were performed at 8–24 h post cell seeding, depending on the cell density and cell growth status. Generally, a 70–90% confluent cell culture was optimal. The DNA/transfection reagent ratio was 1:3 for Lipofectamine 2000 and 1:1.5 for PEI. The DNA was diluted in Opti-MEM and then added to the diluted transfection reagent. After gently mixing and 10–15 min incubation, the DNA complex was added to cells by drops and incubated for 1–2 days at 37 °C.

### 4.4. RNA Isolation and Reverse Transcription

The 22Rv1 cells were washed twice and harvested in 1 × PBS twenty-four hours post-transfection, and total RNA was extracted from the surviving cells using RNeasy Plus Mini Kit (74136, QIAGEN, Dusseldorf, Germany). We treated the mRNA with RapidOut DNA Removal Kit (K2981, Thermo Scientific, Waltham, MA, USA) to remove the trace amount of genomic DNA residue according to the product manual. The purified RNA was then subjected to reverse transcription with High-Capacity cDNA Reverse Transcription Kits (4374967, Applied Biosystems, Waltham, MA, USA). Briefly, 1.5 μg RNA was added into 10 μL of 2× RT Master Mix and made up to the final 20 μL with nuclease-free water. The reactions were incubated at 25 °C for 10 min, followed by 120 min at 37 °C, then were inactivated Reverse Transcriptase by heating to 85 °C for 5 min. The cDNA products were stored at −20 °C or −80 °C and ready for qPCR analysis and NGS sequencing library preparation. For the DiR analysis, the sequence-specific primer BarP6 (CACGATCTGTCCGCACTGCTTGG) was used for reverse transcription, and random primer supplied in the reverse transcription kit was used for reverse transcription for other applications.

### 4.5. Quantitative PCR

We performed RT-qPCR, ChIP-qPCR, and FAIRE-qPCR assays using the AceQ qPCR SYBR Green Master Mix (Q111-03, Vazyme, Nanjing, China) on the thermocyclers Rotor-Gene Q (Qiagen, Dusseldorf, Germany) or LightCycler 96 thermal cycler Instrument (Roche Applied Science, Indianapolis, IN, USA). All the qPCR primer pairs were confirmed to have reasonable specificity and amplification efficiency before qPCR assays, and all the qPCR assays were performed in three technical replications. In the RT-qPCR analysis to analyze the gene expression, the endogenous *ACTB* gene was used for normalization control. For ChIP-qPCR assays, the relative enrichment of the target DNA region was determined by calculating the immunoprecipitation efficiency over input control and then normalized to the control region. In FAIRE qPCR analysis, the enrichment fold of the given region was calculated similarly. Specifically, in the AS-qPCR assay, primers were designed with allele-specific nucleotide placed at the 3′ terminal to enable selective amplification of SNP regions. The DiR-qPCR primers are listed in Table S3, and all the other qPCR primers are listed in Table S4.

### 4.6. DiR-Seq Library Preparation for Illumina Sequencing

The DiR-seq libraries were prepared with two rounds of PCR amplification with cDNA as templates using 2× Phusion Hot Start II High-Fidelity PCR Master Mix (F565L, Thermo Scientific, Waltham, MA, USA). To adapt the 150 bp paired-end sequencing strategy on the Illumina HiSeq X-TEN platform, we divided the 450 bp barcoding region into two amplicons of 271 bp and 270 bp, respectively, in the first round of PCR. During this step, the binding sites of Illumina sequencing primers were introduced at both ends. In the second-round PCR, adaptors for cluster generation and the index sequences were added. Twenty-four sets of primers tiling the flank sequence of the barcoding region in the first round of PCR, in combination with 12 sequencing indexes introduced in the second-round PCR, will enable up to 288 treatments to be analyzed in parallel in one NGS library. The first-round PCR was performed with 2× Phusion Hot Start II High-Fidelity PCR Master Mix using the program: 98 °C for the 30 s of initial denaturation, then 7 cycles of 98 °C for 10 s, 72 °C for 45 s and followed by a final extension at 72 °C for 5 min. The PCR products were purified using 1×VAHTS DNA Clean Beads (N411, Vazyme, Nanjing, China), eluted in 10 μL water, pooled every twenty-four sets of products equally, and then subjected to the second round PCR, which was performed using 2× Phusion HS II HF Master Mix with 1 ng template DNA (98 °C for 30 s, 10 cycles of 98 °C for 10 s, 68 °C for 15 s, 72 °C for 30 s, followed by 72 °C for 5 min). The products were purified using 1×VAHTS DNA Clean Beads and eluted in 15 μL 1×TE buffer. We also subjected the template plasmid pool to NGS library preparation as input control for calculating the expression level. The purified DiR-seq libraries were subjected to 150 bp paired-end sequencing on the Illumina HiSeq X-TEN platform run by Genewiz(NJ, USA), generating about 1 million reads per library. Primers used for DiR-seq library construction are shown in Table S5.

### 4.7. NGS Data Processing

Illumina sequencing raw data were performed quality control using the software FastP (https://github.com/OpenGene/fastp, accessed on 3 December 2018). It is important to note that the 5′ terminal ‘N’ base should not be removed during the cleaning step. The clean Illumina reads were assembled for the paired reads using the software Pandaseq [56], and the sub-libraries were then sorted out using the R package ‘ShortRead’ [57]. Further, we counted the read number of each dinucleotide barcodes using the R package ‘ShortRead’ for each sub-library and normalized the barcode counts by making each sub-library 1 M total reads to eliminate the influence of sequencing depth variation. For each dinucleotide barcode, the expression level was counted by dividing the reads number in cDNA by template DNA. The statistical significance of the expression difference between the two SNP alleles was evaluated with the Two-tailed Student’s *t*-test. All the SNPs determined to have regulatory functions were listed in Table S6.

### 4.8. Luciferase Reporter Assays

DNA fragments bearing the rs684232, rs2955626, rs461251, rs887391, and rs11672691 sites were inserted upstream to the SV40 promoter in the pGL3 Promoter vector. The corresponding DNA fragments sizes are 852 bp, 835 bp, 852 bp, 766 bp, and 766 bp, respectively. The internal Renilla control plasmid pGL4.75 [hRluc/CMV] (E6931, Promega, Fitchburg, WI, USA, RRID:Addgene_24348) and each reporter plasmid were co-transfected into 22Rv1 cells in Nunc™ F96 MicroWell White Polystyrene Plate (136101, Thermo Scientific, Waltham, MA, USA) in a reverse transfection manner using Lipofectamine 2000 Transfection Reagent (11668-019, Invitrogen, Carlsbad, CA, USA) according to the protocol provided by the manufacturer. The luciferase activity was measured with Dual-Glo Luciferase Assay System (E2920, Promega, Fitchburg, WI, USA) at 48 h post-transfection, and the luminescence was acquired using the EnSpire Multimode Plate Reader from PerkinElmer (Manchester, UK). All data were obtained from at least three replicate wells, and statistical analyses were performed with the Two-tailed Student’s *t*-test.

### 4.9. Formaldehyde-Assisted Isolation of Regulatory Elements (FAIRE)

FAIRE assays were performed as previously described [39]. Briefly, cells were fixed with 1% formaldehyde (F8775, Sigma-Aldrich, St. Louis, MO, USA) for 10 min at room temperature, and the fix reaction was quenched with 125 mM glycine (0167, Amresco Radnor, PA, USA). After washing twice with cold PBS, the cells were collected and resuspended in hypotonic lysis buffer (20 mM Tris-HCl, pH 8.0, with 10 mM KCl, 10% glycerol, 2 mM DTT supplied with cOmplete EDTA-free Protease Inhibitor Cocktail) followed by rotation at 4 °C for 30 min. The cell nuclei were washed with cold PBS and then resuspended in 2% SDS lysis buffer (50 mM Tris-HCl, pH 8.1, with 2% SDS, 10 mM EDTA supplied with cOmplete EDTA-free Protease Inhibitor Cocktail) and incubated at 4 °C for 30–60 min. The chromatin was sheared to an average size of 200 bp with a Bioruptor (Bioruptor pico), and the lysate was then cleared by 5 min centrifugation at 13,000× *g* at 4 °C. Chromatin lysate containing 0.5 µg DNA was subjected to twice phenol/chloroform/isoamyl alcohol extraction followed by one chloroform/isoamyl alcohol extraction. The top aqueous layers containing DNA were collected and subjected to ethanol precipitation with the presence of 20 µg of glycogen. The DNA was pelleted and resuspended in 10 mM Tris-HCl (pH 7.4). After being treated with RNase A, the FAIRE DNA and Input DNA were subjected to reverse cross-linking overnight at 65 °C in the presence of proteinase K and purified using 1×VAHTS DNA Clean Beads. The FAIRE DNA was then applied to qPCR analysis to determine the enrichment of the given DNA region in open chromatin or to PCR amplification of SNP regions for Sanger sequencing. All primers are shown in Table S4.

### 4.10. Chromatin Immunoprecipitation (ChIP)

ChIP experiments were performed as described previously [14] with slight modifications. Briefly, chromatin lysate was prepared in the same way as the FAIRE analysis. Immunoprecipitation was performed with antibodies targeting FOXA1 (Santa Cruz Biotechnology, Santa Cruz, CA, USA, Cat# sc-22841, RRID:AB_2104862), H3K27ac (Abcam, Cambridge, UK, Cat# ab4729, RRID:AB_2118291), and H3K4me3 (Abcam, Cambridge, UK, Cat# ab8580, RRID:AB_306649). They were coated onto the Magna ChIP Protein A + G Magnetic Beads (16-663, EMD Millipore, MA, USA) in blocking buffer, which contains 0.5% BSA in IP buffer (20 mM Tris-HCl, pH 8.0, with 2 mM EDTA, 150 mM NaCl, 1% Triton X-100 supplied with cOmplete EDTA-free Protease Inhibitor Cocktail). DNA-protein complex bounded to magnetic beads were washed in turn with wash buffer I (20 mM Tris-HCl, pH 8.0, with 2 mM EDTA, 0.1% SDS, 1% Triton X-100 and 150 mM NaCl), wash buffer II (20 mM Tris-HCl, pH 8.0, with 2 mM EDTA, 0.1% SDS, 1% Triton X-100 and 500 mM NaCl), wash buffer III (10 mM Tris-HCl, pH 8.0, with 1 mM EDTA, 250 mM lithium chloride, 1% deoxycholate and 1% NP-40), and buffer IV (10 mM Tris-HCl, pH 8.0, and 1 mM EDTA) for twice and eluted in extraction buffer (10 mM Tris-HCl, pH 8.0, with 1 mM EDTA and 1% SDS). The complex was incubated with 0.2 mg/mL RNase A (Thermo Scientific, Waltham, MA, USA) for 30 min at 37 °C and subjected to overnight reverse cross-linking at 65 °C with proteinase K (Thermo Scientific, Waltham, MA, USA) and purified using 1×VAHTS DNA Clean Beads. The ChIP DNA was then subjected to qPCR analysis to determine the enrichment of the given DNA region or to PCR amplification of SNP regions to observe the allele selectivity by Sanger sequencing. All primers are shown in Table S4.

### 4.11. Lentiviral Constructs, Lentivirus Production, and Infection

The shRNA constructs targeting *FOXA1* were the same as previously described [15], and the *VPS53*, *FAM57A*, and *GEMIN4* shRNA in pLKO.1-puro were designed according to the validated shRNA clones in MISSION^®^ shRNA Library (Sigma-Aldrich, St. Louis, MO, USA). Detailed information on these shRNA constructs is provided in Table S7. Lentivirus expressing given shRNA was produced with the third-generation packaging system in Lenti-X 293T cells (Clontech). Briefly, 70–80% confluent Lenti-X 293T cells in 6-well plates were transfected with 3 µg shRNA plasmid, 1 μg pVSVG (envelope plasmid, RRID:Addgene_85140), 1 μg pMDLg/pRRE (packaging plasmid, RRID:Addgene_12251), and 1μg pRSV-Rev (packaging plasmid, RRID:Addgene_12253) in a 3:1:1:1 ratio using PEI (408727, Sigma, St. Louis, MO, USA) in an FBS and antibiotics free medium [38]. The medium was replaced with fresh complete DMEM containing 10% FBS (Gibco, New York, NY, USA) and 1% Penicillin-Streptomycin (Sigma-Aldrich, St. Louis, MO, USA) after 4–8 h, and the virus supernatant was collected every 12 h for up to six times. The supernatant containing viral particles was cleared by centrifugation at 1000× *g* and passed through a 0.45 μm filter unit (Millipore) and then stored at −80 °C in aliquots or used directly for subsequent experiments.

For viral infection, target cells were seeded in a 6-well plate and grown for 16–24 h until they reach 60–70% confluence. The growth medium was replaced with the virus supernatant supplied with 8 μg/mL polybrene (Sigma-Aldrich, St. Louis, MO, USA). Twenty-four hours later, the virus-containing medium was replaced with the complete medium with puromycin (Sigma, St. Louis, MO, USA) at 4 μg/mL for 22Rv1. When control cells without virus infection were all dead, the surviving cells were split and cultured in the same growth medium. After three days, the cells were collected for RNA preparation and RT-qPCR gene expression quantification. The most efficient shRNA for each gene was selected for subsequent analysis, including cell proliferation assays and cell colony formation assays.

### 4.12. rs684232 Knockout Using CRISPR/Cas9

We designed the gRNA sequences that guide Cas9 cleavage on both alleles precisely to the left of the rs684232 site. The oligos were annealed and jointed with the BbsI (FD1014, Thermo Scientific, Waltham, MA, USA) digested pSpCas9(BB)-2A-Puro (PX459) V2.0 (RRID:Addgene_62988) [58]. For negative control, the sgRNA was designed to target a non-Mammalian sequence. All the oligos sequences for gRNA sequences are listed in Table S7. The CRISPR plasmids were transfected into 22Rv1 cells at 70% confluence in a 12-well plate using Lipofectamine 2000 Transfection Reagent (11668-019, Invitrogen, Carlsbad, CA, USA) according to the manufacturer’s instructions. After 24 h, the medium was replaced with fresh medium supplied with puromycin at a final concentration of 4 μg/mL. When non-transfected cells all died, the surviving cells were split and cultured in the complete medium and subjected to editing efficiency evaluation by getPCR analysis. The surviving cells were then trypsinized and seeded into 96-well plates at a dilution to have less than one cell at each well. The single-cell clones were propagated for 1 to 2 months and screened through the getPCR method. The clones that had desired rs684232 mutation were further genotyped through the Sanger sequencing method.

### 4.13. Genome Editing Efficiency Determination and Single-Cell Clone Screening

To detect genome editing, we performed getPCR assays as previously described [59]. Briefly, for determining the genome editing efficiency, the tested primer was designed with four watching nucleotides. The getPCR was performed using 7.5 μL AceQ qPCR SYBR Green Master Mix (Vazyme, Nanjing, China) on Roche LightCycler96. While screening the single-cell clones that happened anticipated modification, we used the watching primers with their 3’ end located on the rs684232 site. The control amplification was designed 200 bp away from the cutting site, which was used for normalization purposes in calculating the percentage of wild-type DNA in the edited genomic DNA. The primers used in getPCR experiments are listed in Table S4.

### 4.14. Cell Viability and Proliferation Assays

To investigate the cell proliferation, the 22Rv1 cells that underwent infection with lentiviral particles or single-cell clones with rs684232 site deleted through genome editing were counted and seeded into 96-well cell culture plates at 5 × 103 per well. Cell viability and proliferation were measured with a CCK-8 kit (MA0218, Meilun, Dalian, China), and the optical density at 450 nm was acquired on an M200 PRO multimode plate reader (Tecan, Sunnyvale, USA) every 24 h. The results were obtained from three independent experiments, and the statistical significance was calculated with the Two-tailed Student’s *t*-test.

### 4.15. Colony-Forming Assay

Cells were trypsinized into single cells and seeded into 6-well plates with 1500 cells per well. The medium was replaced with fresh medium every three days. After 15–20 days, the medium was discarded, and cells were washed twice with 1 mL cold 1×PBS carefully. After fixation with 2 mL 100% methanol for 30 min, the cells were further stained with 2 mL of 0.05% Crystal Violet staining solution (HY-B0324A, MCE, Shanghai, China) for 30 min. The cells were washed twice with deionized water and dried overnight and lysed with 1% SDS in 0.2 N NaOH for 1 h, and the optical density at 570 nm was acquired on an M200 PRO multimode plate reader (Tecan). A blank well without cell was set as a control to minus the background staining. All data came from three replicate wells, and the statistical significance was calculated with the Two-tailed Student’s *t*-test.

### 4.16. Wound Healing Assays

The wound-healing assay was performed as previously described [60]. Briefly, the 22Rv1 cells were seeded in a 6-well plate in the serum-free medium at a density that made 90% confluence 12 h later. Made a scratch wound on the cell monolayer using a 200 μL pipette tip and washed the cells three times with fresh medium to remove the debris and smooth the edge. The cells were grown in a complete medium containing 10% FBS, and the wound healing process was imaged (10×) using an inverted fluorescence microscope (Olympus, Tokyo, Japan) every 24 h. The wound closure area in each well was analyzed using ImageJ software (ImageJ, RRID:SCR_003070).

### 4.17. Statistical Analysis

For the DiR-qPCR, DiR-seq, RT-qPCR, ChIP-qPCR, and FAIRE-qPCR analysis as well as for the evaluation of cell proliferation, cell migration, and colony formation, we used the Two-tailed Student’s *t*-test.

The transcriptome data was downloaded from The Cancer Genome Atlas (TCGA, The Cancer Genome Atlas, RRID:SCR_003193) database using the R package “TCGAbiolinks” (TCGAbiolinks, RRID:SCR_017683) for differential gene expression analysis for prostate cancer tissues and Para-cancerous tissues. The GDC.h38 GENCODE v22 GTF file for gene annotation was used to match the data file to TCGA ID, and the transcriptome counts data were further processed with the R package “Deseq2” (DESeq2, RRID:SCR_015687). The differential expression levels of *VPS53*, *FAM57A*, and *GEMIN4* gene in prostate cancer tissues and Para-cancerous tissues were visualized as violin box plots using “ggplot2” (ggplot2, RRID:SCR_014601). We used the Mann-Whitney U test to evaluate the statistical significance of gene expression differences between normal and tumor tissues.

For the correlation analysis of gene expression in tissues of prostate cancer or pan-cancer of 33 cancer types, we obtained the gene expression RNA-seq data as “TOIL RSEM tpm” file from the TCGA Pan-Cancer (PANCAN) cohort and annotated it with the gtf file “genecode. v23.annotation”. The cancerous tissues were then extracted out and subjected to calculating the correlation coefficient and *p*-value using the “ggplot2” package.

For Kaplan-Meier survival analysis in prostate cancer patients or Pan-Cancer of 33 cancer types, we obtained the integrated TCGA Pan-Cancer Clinical Data from Liu’s work [61] and merged to the gene expression matrix of Pan-Cancer tissues aforementioned. We then used the R package “survival” (survival, RRID:SCR_021137) and “survminer” (survminer, RRID:SCR_021094) to perform Kaplan–Meier survival analysis and visualization. Patients were sub-grouped based on the optimal cut-off point determined using the “survminer” R package. We used the Cox proportional hazards model to assess the hazard ratio (HR) and log-rank test to assess the statistical significance between the two groups of patients. We used R-4.0.2 for running the R packages.

## Data Availability

The raw sequence data generated using the Illumina Hiseq-PE150 platform for DiR-seq and 10-nucleotide tag reporter assay have been publicly available in the Gene Expression Omnibus (GEO) database under the accession number GSE165765.

## Supplementary Materials

The following are available online at https://www.mdpi.com/article/10.3390/ijms22168792/s1.
