# Supplementary material for: Parallel Reporter Assays Identify Altered Regulatory Role of rs684232 in Leading to Prostate Cancer Predisposition
Source: Int J Mol Sci. 2021 Aug 16;22(16):8792. doi: 10.3390/ijms22168792 (PMC8395720; doi:10.3390/ijms22168792)
Supplement: Supplementary file 1 [file ijms-22-08792-s001.zip › Supplementary figures.pdf]

Figure S1 Allele-specific chromatin open status of 13 heterogeneous SNPs in 22Rv1.

Figure S2 Gene regulatory function of rs887391 and rs11672691.

Figure S3 The Genotype of the rs684232 edited 22Rv1 cell.

Figure S4 Chromatin status of rs2955626 and rs461251.

Figure S5 Luciferase reporter assay of rs2295626 and rs461251.

Figure S6 Genes expression correlation between *VPS53, FAM57A,* and GEMIN4.

Figure S7 Pan-Cancer Kaplan-Meier Survival analysis of *VPS53, FAM57A*, and *GEMIN4* genes.

Figure S8 TCGA prostate cancer disease-free interval analysis of *VPS53, FAM57A*, and *GEMIN4* genes.

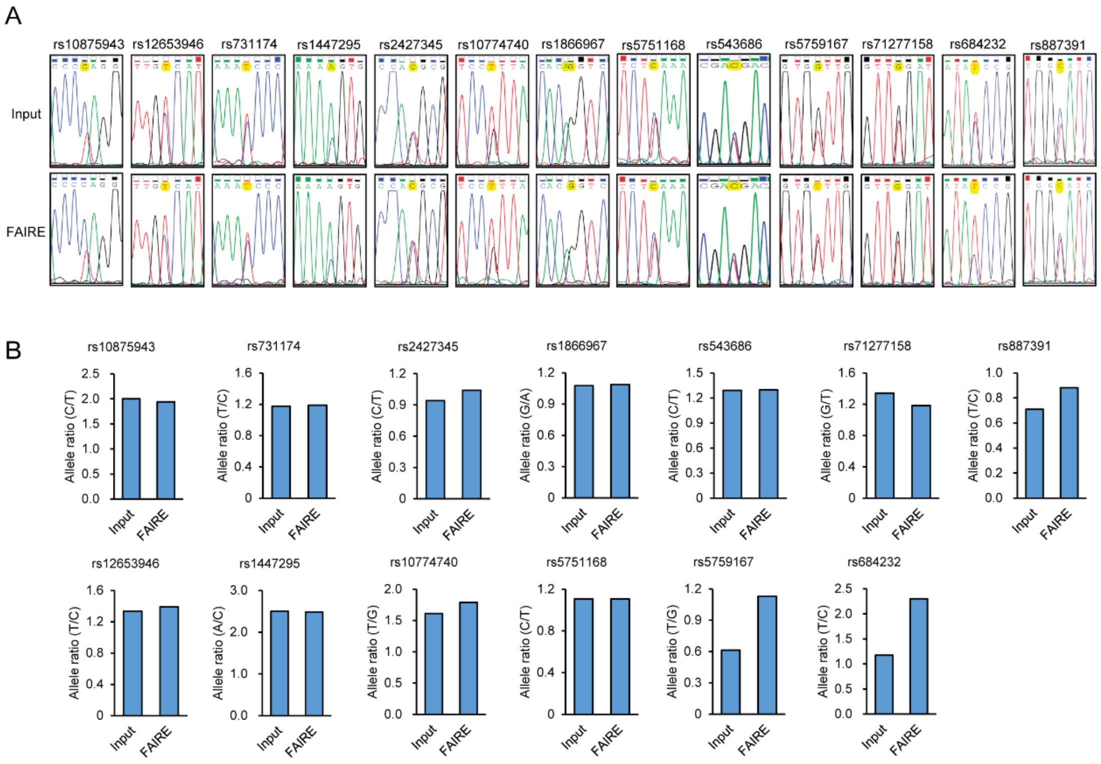

Figure S1 Allele-specific chromatin open status of 13 heterozygous SNPs in 22Rv1.

(**A**) Sanger sequencing chromatography of FAIRE and input DNA for the 13 heterozygous SNPs in the 22Rv1 cells. The SNP positions are highlighted with a yellow circle background.

(**B**) The allele ratios for the 13 heterogeneous SNPs in FAIRE and input DNA, quantified by the EditR 1.0.9 online software[1].

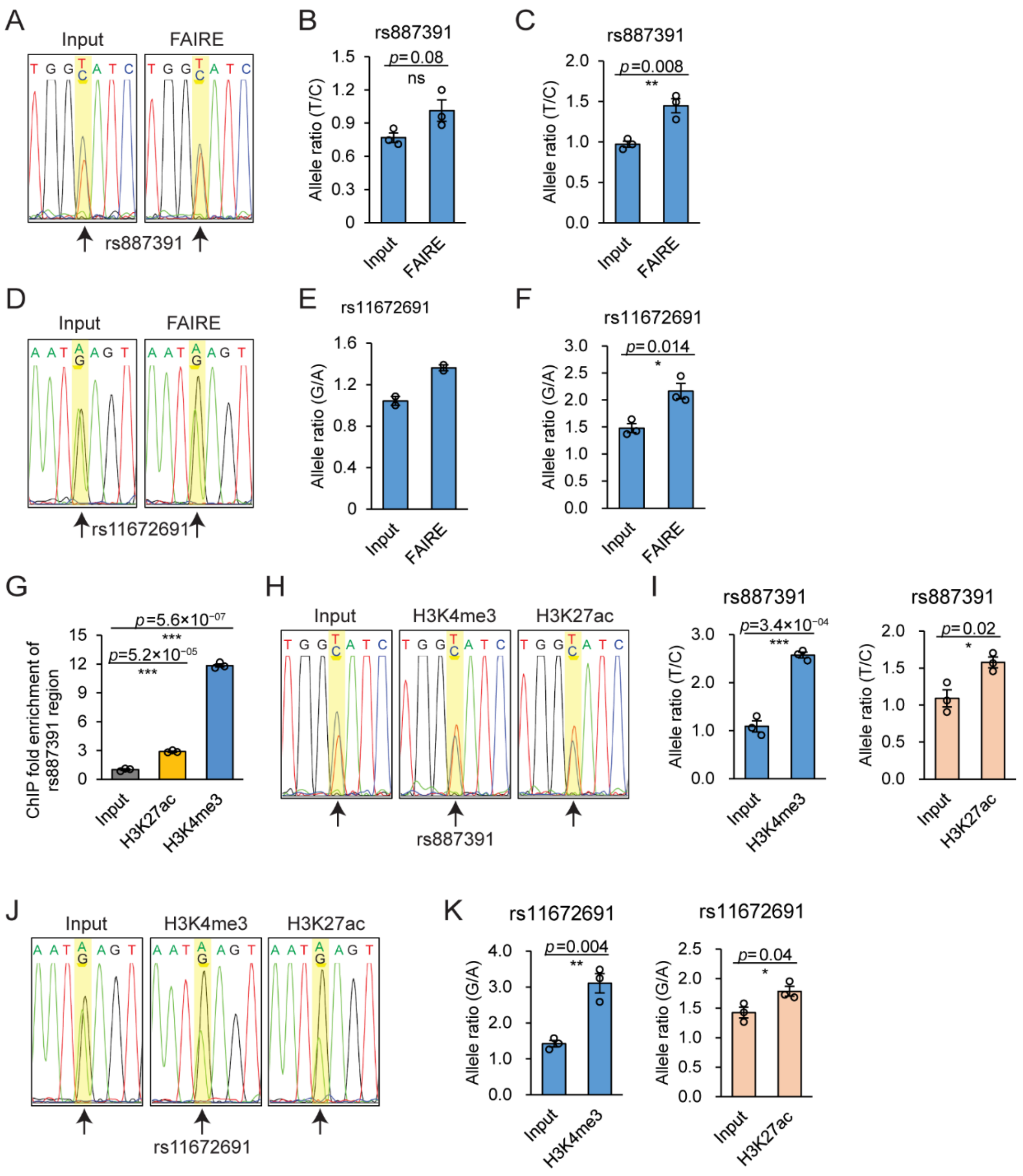

Figure S2 Gene regulatory function of rs887391 and rs11672691.

(**A**) Sanger sequencing chromatography of FAIRE DNA and input DNA for rs887391 in 22Rv1 cells. The position of rs887391 is highlighted with a yellow square and arrow. (**B**) The allele ratios of rs887391 in FAIRE and input DNA were quantified by the EditR [1]. (**C**) Allele-specific enrichment of rs887391 in FAIRE DNA in 22Rv1 cells determined by AS-qPCR. Mean ± SEM of three independent experiments. (**D**) Sanger sequencing chromatography of FAIRE DNA and input DNA for rs11672691 in 22Rv1 cells. The position of rs11672691 is highlighted with a yellow square and arrow. (**E**) The allele ratios of rs11672691 in FAIRE and input DNA were quantified by the EditR [1]. Mean ± SEM of two independent experiments. (**F**) Allele-specific

enrichment of rs11672691 in FAIRE DNA in 22Rv1 cells determined by AS-qPCR. Mean ± SEM of three independent experiments. (**G**) Enrichment of rs887391 region in the H3K27ac and H3K4me3 ChIP DNA in 22Rv1 determined through qPCR analysis. The rs11672691 site is 36bp upstream to the rs684232. Mean ± SD of three technical replicates. (**H**) Sanger sequencing chromatography of H3K27ac and H3K4me3 ChIP DNA around the rs887391 site in 22Rv1 cells. The position of rs887391 is highlighted with a yellow square and arrow. (**I**) Allelic ratios of rs887391 in H3K27ac and H3K4me3 ChIP DNA determined by AS-qPCR. Mean ± SD of three technical replicates. (**J**) Sanger sequencing chromatography of H3K27ac and H3K4me3 ChIP DNA around rs11672691 site in 22Rv1 cells. The position of rs11672691 is highlighted with a yellow square and arrow. (**K**) Allelic ratios of rs11672691 in H3K27ac and H3K4me3 ChIP DNA were determined by AS-qPCR. Mean ± SD of three technical replicates. *p<0.05, **p<0.01, ***p<0.001, two-tailed Student's t-test.

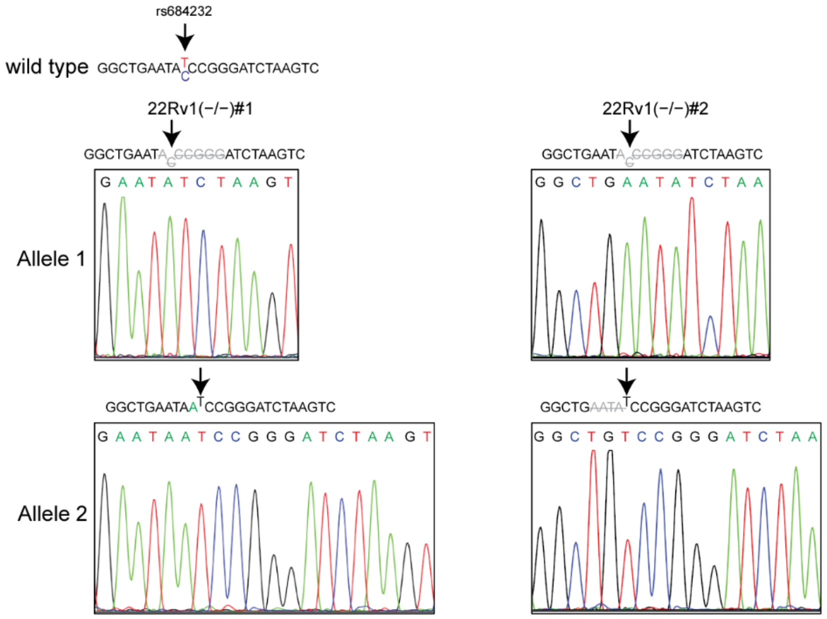

Figure S3 The Genotype of the rs684232 edited 22Rv1 cell.

Two single-cell clones with rs684232 site indel mutation were obtained using the CRISPR/Cas9 technique. rs684232 knocked out cell lines. The genotypes were determined using Sanger sequencing of the rs684232 region cloned into T-vector. For the 22Rv1(−/−) #1 clone, allele 1 has six nucleotides deleted, and allele 2 has one "A" nucleotide inserted in front of rs684232-T. For the 22Rv1(−/−) #2 clone, allele 1 has six nucleotides lost, and allele 2 has four nucleotides deleted.

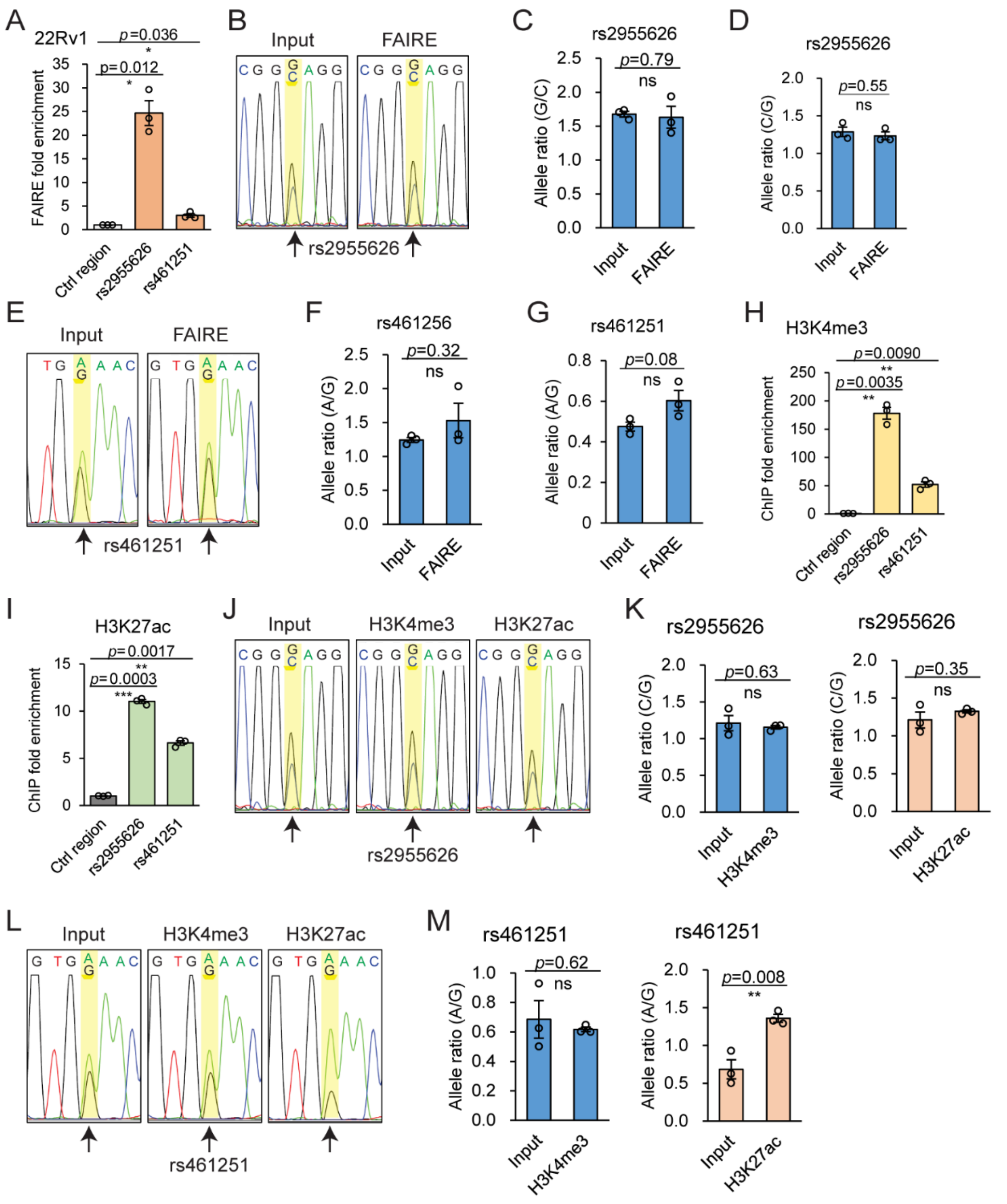

Figure S4 Chromatin status of rs2955626 and rs461251.

(**A**) FAIRE qPCR analysis in 22Rv1 cells to evaluate chromatin open status at rs2955626 and rs461251. Mean ± SEM of three independent experiments. (**B**) Sanger sequencing chromatography of FAIRE and Input DNA around rs2955626 site in 22Rv1 cells. The position of rs2955626 is highlighted with a yellow square and arrow. (**C**) The allele ratios of rs2955626 in FAIRE and input DNA were quantified by the EditR [1]. Mean ± SEM of three independent experiments. (**D**) Allele-specific enrichment of rs2955626 in FAIRE DNA in 22Rv1 cells determined by AS-qPCR. Mean ± SEM of three independent experiments. (**E**) Sanger

sequencing chromatography of FAIRE and Input DNA around rs461251 site in 22Rv1 cells. The position of rs461251 is highlighted with a yellow square and arrow. (**F**) The allele ratios of rs461251 in FAIRE and input DNA were quantified by the EditR [1]. Mean ± SEM of three independent experiments. (**G**) Allele-specific enrichment of rs461251 in FAIRE DNA in 22Rv1 cells determined by AS-qPCR. Mean ± SEM of three independent experiments. (**H**) ChIP qPCR analysis to evaluate the enrichment of rs2955626 and rs461251 sites in histone H3K4me3 modification in 22Rv1 cells. Mean ± SD of three technical replicates. (**I**) ChIP qPCR analysis to evaluate the enrichment of rs2955626 and rs461251 sites in histone H3K27ac modification in 22Rv1 cells. Mean ± SD of three technical replicates. (**J**) Sanger sequencing chromatography of H3K27ac and H3K4me3 ChIP DNA around rs2955626 site in 22Rv1 cells. The position of rs2955626 is highlighted with yellow square and arrow. (**K**) Allelic ratios of rs2955626 in H3K27ac and H3K4me3 ChIP DNA were determined by AS-qPCR. Mean ± SD of three technical replicates. (**L**) Sanger sequencing chromatography of H3K27ac and H3K4me3 ChIP DNA around rs461251 site in 22Rv1 cells. The position of rs461251 is highlighted with yellow square and arrow. (**M**) Allelic ratios of rs461251 in H3K27ac and H3K4me3 ChIP DNA were determined by AS-qPCR. Mean ± SD of three technical replicates. *$p<0.05$, **$p<0.01$, ***$p<0.001$, two-tailed Student's t-test.

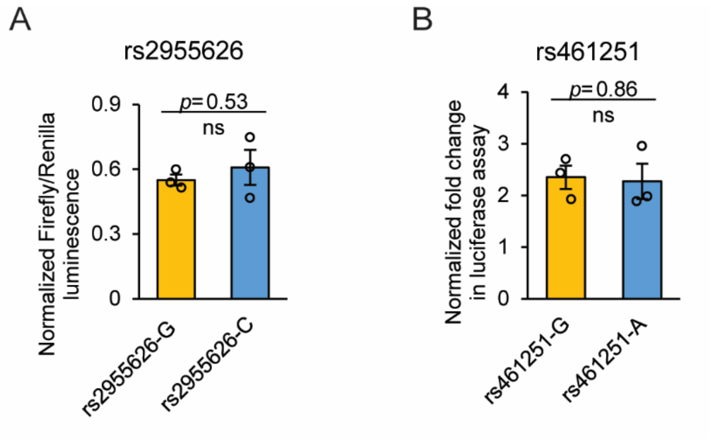

Figure S5 Luciferase reporter assay of rs2295626 and rs461251.

(**A**) Luciferase reporter assay of rs2295626 region in 22Rv1 cells. (**B**) Luciferase reporter assay of rs461251 region in 22Rv1 cells. *P*-value was calculated with the two-tailed Student's t-test.

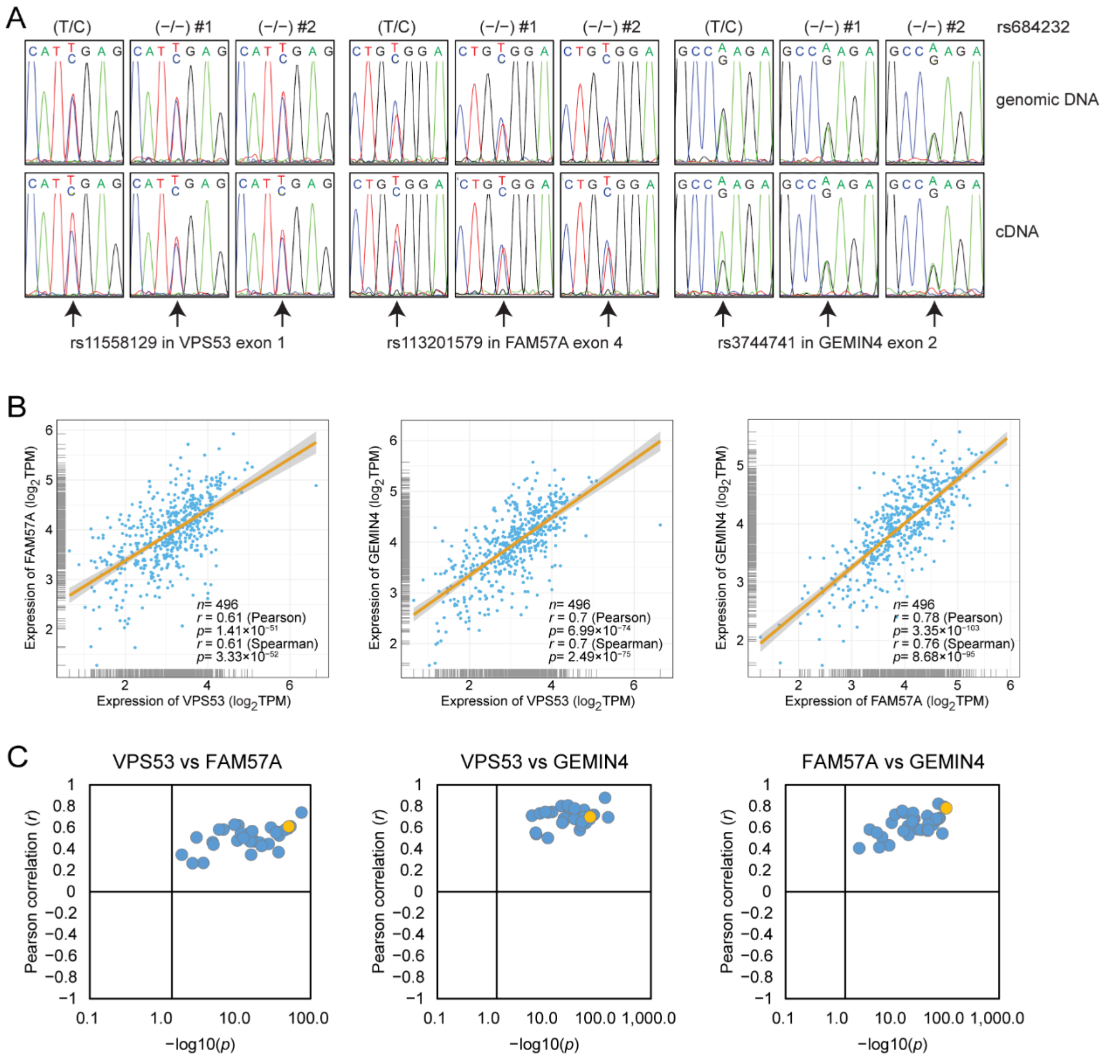

Figure S6 Genes expression correlation between *VPS53*, *FAM57A*, and *GEMIN4*.

(**A**) Sanger sequencing chromatography of cDNA around the heterozygous SNPs in exons of given genes in genome-edited 22Rv1cells. The positions of the three SNP sites are highlighted with arrows. (**B**) Pairwise gene expression correlation between *VPS53*, *FAM57A*, and *GEMIN4* in prostate cancer tissues from TCGA. Every dot represents one clinical sample. Correlation coefficient (*r*) values and *P* values were calculated from the Pearson correlation and Spearman correlation analysis. (**C**) Pairwise gene expression correlation between *VPS53*, *FAM57A*, and *GEMIN4* in 33 kinds of cancer tissues from TCGA. The Pearson correlation coefficient value of each cancer type was plotted vs. the -log10 of *P*-value. The dot in yellow represents the PRAD. Correlation coefficient (*r*) values and *P* values were from the Pearson correlation analysis.

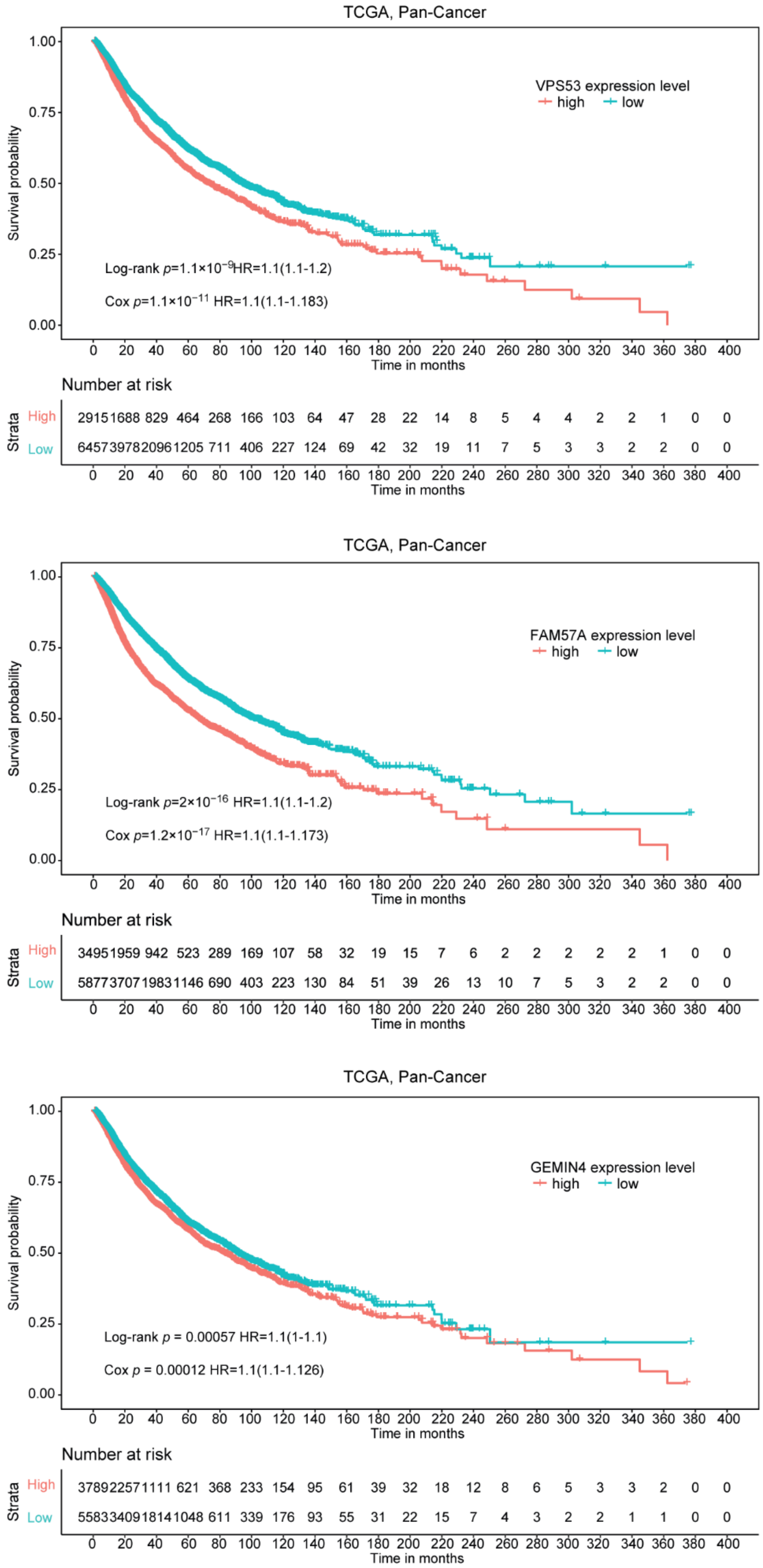

Figure S7 Pan-Cancer Kaplan-Meier Survival analysis of *VPS53, FAM57A,* and *GEMIN4*

genes. The 9372 TCGA cancer patients of 33 cancer types were stratified into two groups according to the gene expression level of *VPS53, FAM57A*, and *GEMIN4*, respectively. Higher levels of *VPS53, FAM57A,* and *GEMIN4* correlate with decreased overall survival probability. *P* values determined by a log-rank test and Cox regression analysis, with a 95% confidence interval.

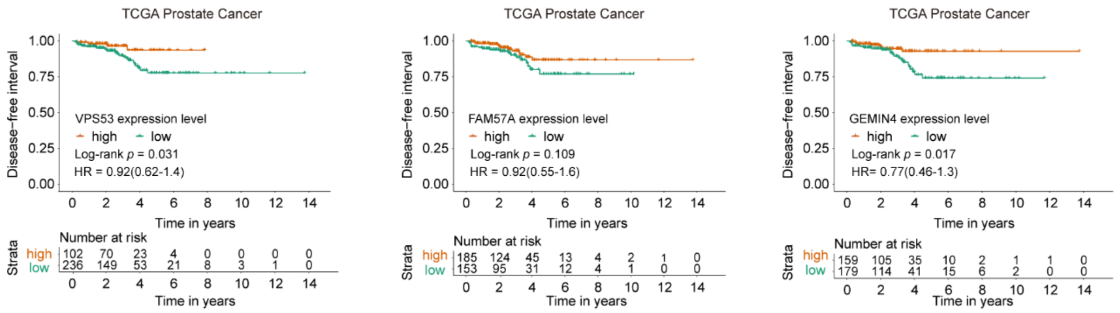

Figure S8 TCGA prostate cancer disease-free interval analysis of *VPS53*, *FAM57A*, and *GEMIN4* genes. Prostate cancer patients were stratified into two groups according to the expression level of *VPS53* (strata point=2.3), *FAM57A* (strata point=3.67), and *GEMIN4* (strata point=4.6). Lower levels of *VPS53*, *FAM57A*, and *GEMIN4* correlate with an increased risk of recurrence. *P* values calculated by the log-rank test, with a 95% confidence interval.

References

1. Kluesner, M.G. et al. EditR: A Method to Quantify Base Editing from Sanger Sequencing. *CRISPR J* **1**, 239-250 (2018).
